# Inequalities in neighbourhood socioeconomic characteristics: potential evidence-base for neighbourhood health planning

**DOI:** 10.1186/1476-072X-4-20

**Published:** 2005-08-10

**Authors:** Agricola Odoi, Ron Wray, Marion Emo, Stephen Birch, Brian Hutchison, John Eyles, Tom Abernathy

**Affiliations:** 1 Department of Clinical Epidemiology and Biostatistics, McMaster University, Hamilton, Ontario, Canada; 2 Formerly with Hamilton District Health Council, Hamilton, Ontario, Canada; 3 Canadian Institute for Health Information, Toronto, Ontario, Canada; 4 Centre for Health Economics and Policy Analysis, McMaster University, Hamilton, Ontario, Canada; 5 School of Geography and Geology, McMaster University & McMaster Institute of Environment & Health, Hamilton, Ontario, Canada

## Abstract

**Background:**

Population health planning aims to improve the health of the entire population and to reduce health inequities among population groups. Socioeconomic factors are increasingly being recognized as major determinants of many aspects of health and causes of health inequities. Knowledge of socioeconomic characteristics of neighbourhoods is necessary to identify their unique health needs and enhance identification of socioeconomically disadvantaged populations. Careful integration of this knowledge into health planning activities is necessary to ensure that health planning and service provision are tailored to unique neighbourhood population health needs. In this study, we identify unique neighbourhood socioeconomic characteristics and classify the neighbourhoods based on these characteristics. Principal components analysis (PCA) of 18 socioeconomic variables was used to identify the principal components explaining most of the variation in socioeconomic characteristics across the neighbourhoods. Cluster analysis was used to classify neighbourhoods based on their socioeconomic characteristics.

**Results:**

Results of the PCA and cluster analysis were similar but the latter were more objective and easier to interpret. Five neighbourhood types with distinguishing socioeconomic and demographic characteristics were identified. The methodology provides a more complete picture of the neighbourhood socioeconomic characteristics than when a single variable (e.g. income) is used to classify neighbourhoods.

**Conclusion:**

Cluster analysis is useful for generating neighbourhood population socioeconomic and demographic characteristics that can be useful in guiding neighbourhood health planning and service provision. This study is the first of a series of studies designed to investigate health inequalities at the neighbourhood level with a view to providing evidence-base for health planners, service providers and policy makers to help address health inequity issues at the neighbourhood level. Subsequent studies will investigate inequalities in health outcomes both within and across the neighbourhood types identified in the current study.

## Background

Traditional health planning has typically focused on the practice and delivery of health care services. Population health planning, on the other hand, aims to improve the health of the entire population and to reduce health inequities among population groups [1]. The health of a population is influenced by several factors including but not limited to socioeconomic status [2], social support networks [3] education [4], ethnicity [5], employment [6], working conditions [7-10], physical environment [11], personal health behaviours [12,13], health care services [14] and individual coping skills [15,16]. Therefore, health planning, policy and interventions need to take into consideration not only the health care services, but also these broad determinants of health.

Since socioeconomic and demographic characteristics are important determinants of population health, adopting a population health approach to health planning at the neighbourhood level requires improved knowledge of the distribution of population socioeconomic and demographic characteristics at this level. Globally, there is an increasing interest in understanding the relationship between neighbourhood of residence and health of the population [17-19]. To this end, some researchers have suggested that improving the health of those living in the worst areas calls for systematically exploring area differences to inform social and health policy [20].

Currently, the lowest geographical level at which most health-planning data in Canada are analyzed is the municipal (city) level. Obviously, the use of such a large unit of analysis limits the ability to identify specific population characteristics as well as health variations and needs at the lower levels. The implication is that disparities in health outcomes and access to health care services across population sub-groups at these lower levels are unclear. Moreover, most large cities have diverse populations [21-24]; therefore the neighbourhoods within them have diverse socioeconomic and demographic characteristics that may influence neighbourhood population health outcomes and therefore health needs [25-27]. A number of studies have shown the extent and causes of neighbourhood socioeconomic inequalities [21-23,28-31]. In Canada, there is evidence that neighbourhood socioeconomic inequality has been rising since 1970 [32,33]. Moreover, numerous studies have reported associations between neighbourhood socioeconomic characteristics and various health outcomes [34-42]. Therefore, taking into consideration the diverse socioeconomic and demographic characteristics of the different neighbourhoods during health planning would ensure that planning and health services are tailored to the unique needs of the local residents of each neighbourhood.

Studies of geographical distribution of determinants of health have mainly used one of three approaches. The first involves either production of a single map showing the spatial distribution of a single variable (determinant of health) or production of a series of maps each showing the distribution of a single determinant of health [43-45]. The limitations of this method are that only one determinant can be assessed at a time(if a single variable approach is used) and assumes each determinant is independent of other determinants (if a series of maps is used). Moreover, when a series of maps is used, interpretation may be difficult. In the second approach, a composite index is created from combining two or more variables [46-48]. Although this approach mitigates the limitations of approach 1 and is effective in highlighting areas considered to be "high-risk", its drawback is that specific population characteristics (e.g. education, ethnicity, income, etc) are rolled into an index so that one cannot identify distinct characteristics (with respect to these variables) attributable to specific geographical areas. A third analytical approach uses factor analysis (or principal components analysis) to investigate several determinants [49,50]. This approach is a data reduction technique used to reduce the dimensionality of the data from several variables to a few factors (or principal components) that explain most of the variability in the original data. The current study uses principal components analysis and cluster analysis and Geographical Information Systems (GIS) to mitigate the limitations of the above approaches.

The objectives of this study were to use multivariate statistical techniques to identify the socioeconomic and demographic characteristics of neighbourhoods in the city of Hamilton, Ontario, Canada; and to classify the neighbourhoods based on similarities of these characteristics. Potential applications of the methodology in needs-based neighbourhood population health planning, service delivery, and policy development are proposed.

## Methodology

### Study area and geographical scale of analysis

The study was carried out in the city of Hamilton, Ontario, Canada. The city has a population of over 490,000 people and spans over 1,117 square kilometres [51]. A number of factors were considered in selecting the appropriate level of geography for the study. These included homogeneity of socioeconomic variables within the geographical unit; large enough population size to minimize the "small number problem"; data availability; acceptability by health planners and health service providers; and stability of the boundaries over time for maximum temporal data comparability for future analyses.

Based on the above criteria, census tracts were chosen as the most appropriate level of geography for the analysis. A census tract (CT) is a small, relatively stable geographic unit usually having a population of 2,500 to 8,000 persons with an average of approximately 4,000 [52]. There are 132 CTs in the City of Hamilton. Census tracts are used in this study to represent neighbourhoods because CTs have 'neighbourhood-like' characteristics due to their homogeneity with respect to socioeconomic and demographic characteristics [53]. Therefore, throughout this paper, CTs and neighbourhoods are used interchangeably. There were several advantages of adopting this as the level of geography for analysis and future health planning: (i) Most census data are reported at this level of geography; (ii) Administrative health data can easily be aggregated to this level, if the postal codes of the health care recipients are known (this is because, in Canada, the postal code areas are smaller than CTs are so postal code data can easily be aggregated to the CT level); (iii) Census tracts are homogeneous with respect to socioeconomic and demographic characteristics; (iv) Their population sizes are large enough to allow calculation of relatively stable rates of most health events; (v) The boundaries of the CTs follow permanent and easily recognizable physical features and changes to their boundaries are discouraged to maintain maximum data comparability over time [52].

### Data source and variable selection

Socioeconomic and demographic data for 833 dissemination areas (DA) in the city of Hamilton, Ontario, Canada, were extracted from the 2001 Canadian census data [52]. A DA is a small area composed of one or more neighbouring blocks and is the smallest standard geographic area for which all census data are disseminated in Canada [52]. The data were then aggregated to the CT level at which all analyses were performed. The variables used in the analyses were chosen based on their usefulness as determinants of health [54], reliability, and availability at the DA level [55]. Care was taken to include as many socioeconomic and demographic variables as possible in order to enhance the highest statistical differentiation between the CTs [55]. A total of 18 variables measuring the following characteristics were included in the analyses: demographic structure, social status, economic status, ethnicity, aboriginal status, and housing (see Table 1 for a complete list of variables).

**Table 1:** Variables included in the Hamilton neighbourhood analysis. Most definitions were adopted from Statistics Canada health region peer groups Study [55]. Those not adopted from the Statistics Canada study are: median income, married, live alone, population under 20 and non-official language population.

**Variable name**	**Definition**
Persons with less than grade 9 education	Percentage of the pop 20 years and over with less than grade 9 education
New immigrants	The percentage of immigrants who came to Canada from 1996 to 2001
Visible minority	Percentage of the population belonging to a visible minority group. As defined by the employment equity act (1986), visible minorities are persons (other than Aboriginal people) who are non-Caucasian in race or non-white in colour.
Aboriginal persons	Percentage of population reporting at least one Aboriginal origin (North American Indian, Métis or Inuit)
Median income	Median personal income for persons aged 15 and over, from all sources.
Government transfer income	Percentage of all income that came from government transfers (e.g., Canadian pension plan (CPP), guaranteed income supplement (GIS), old age security, etc.) for the population 15 years of age and older.
Incidence of low income (LICO)	Percentage of persons in economic families and unattached individuals with 2000 incomes below the Statistics Canada low-income cut-off (LICO). The cut-offs represent levels of income where people spend disproportionate amounts of money for food, shelter, and clothing. LICOs are based on family size and degree of urbanization; cut-offs are updated to account for changes in the consumer price index.
Non-official language pop	Percentage of the population not speaking any of the two official languages.
Unemployment rate	Total number of unemployed individuals 15 and older divided by the total number of individuals 15 and older participating in the labour force.
Average dwelling value	Average expected value of an owner-occupied, non-farm, non-reserve dwelling (including the value of the land the dwelling is on) at the time of the census
Owner-occupied dwellings	Percentage of dwellings in which the owner also lives. Band housing and collective dwellings (i.e. rooming houses, nursing homes, military camps etc.) Are excluded from both numerator and denominator.
Population under 20 years old	Percentage of the population under the age of 20 years
Population 65 years or older	Percentage of the population aged 65 years or older
Single-parent Families	Percentage of single-parent families among all census families living in private households. A census family refers to a married or common-law couple or lone parent with at least one never-married son or daughter living in the same household.
Married	Percent of legally married persons 15 & over
Live alone	Percent of persons living alone in private households
Internal migrant mobility	Percent of the population that lived in a different Canadian municipality at the time of the previous census. Excludes Canadians in households outside Canada (military & government personnel)
Population density	Total population of a census tract divided by its area in Km^2^

#### Statistical analyses

##### Variable standardization and correlation analysis

All statistical analyses were performed in STATA [56]. To overcome the impact of differing variances and different scales of measurements (e.g. dollars vs percentages), all variables used in the analyses were standardized to mean 0 and unit variances [57]. Had standardization not been performed, variables with high variances would unduly dominate the results of the analyses. A correlation matrix was constructed to explore relationships among the variables.

##### Principal components analysis

Principal component analysis (PCA) was used to reduce the dimensionality of data and to investigate the nature of the relationships among the CTs with the main objective of isolating the general features that best describe the variations in the data. Using this method, 18 inter-correlated socioeconomic and demographic factors were reduced to 5 principal components each of which represented different aspects of the original data. Kaiser criterion (eigenvalue one test) was used to guide the decision on the number of principal components to retain; all components with eigenvalues equal to or less than 1 were not retained since they explained variations equal to or less than any one of the original variables [58,59]. To maximize the variance of factor loadings and therefore aid the separation of CTs (or neighbourhoods) into homogeneous groups, varimax rotation was used [60].

##### Cluster analysis

Cluster analysis is a multivariate statistical technique used to organize observations into groups (or clusters) such that observations within a cluster have a high degree of similarity (or natural associations) among themselves while the clusters are relatively distinct from each other [57]. There are many different definitions of a cluster [57]. For the purpose of this study, we define a cluster as a set of entities that are alike.

There are two major classifications of cluster analysis techniques: hierarchical and non-hierarchical (or partition) techniques. This study adopted the partition cluster analysis, using k-means clustering methodology, to group CTs (or neighbourhoods) based on socioeconomic and demographic characteristics into clusters or neighbourhoods types. Using this methodology, the user specifies the number of clusters, say x, to create. These x clusters are formed through an iterative process. The algorithm begins with x seed values which act as the initial x group means. Observations are then assigned to the nearest group seed. After all observations have been assigned, cluster means are computed for each group. The initial cluster seeds are then replaced by their respective cluster means. The observations are then re-assigned to the nearest cluster mean. These steps continue until no observations change groups (clusters).

The optimum number of groups or clusters or "neighbourhood types" to be identified was decided upon using Calinski-Harabasz pseudo-F test [61] as well as the distribution of the CTs within the cluster. Five clusters (groups) were found to provide the most optimal separation of the CTs within the clusters. This would allow a reasonable number of relatively homogeneous CTs (or neighbourhoods) per group (or neighbourhood type). Formation of more groups (neighbourhood types) resulted in some with too few CTs whereas formation of fewer groups resulted in loss of homogeneity within the groups.

The similarity (or dissimilarity) measure (also known as distance measure) used for the classification of the CTs was Minkowski distance metrics with argument 2 (L2) [57]. This measure, commonly known as euclidean distance, was calculated as follows:



where: p (in this case 18) is the number of variables included in the cluster analysis; x_ik _and x_jk _are the values of variable k for CTs i and j respectively. The summations are over the p (or 18) variables involved in the cluster analysis. The 5 initial cluster centres were obtained randomly from among the CTs or neighbourhoods in the study area. For reproducibility a random number seed was applied before the 5 CTs were randomly chosen.

Since neighbourhoods belonging to the same group have certain socioeconomic and demographics characteristics in common, the resultant grouping provides useful insights into understanding the socioeconomic and demographic characteristics of each group (or neighbourhood type).

##### Cartographic manipulations

All cartographic manipulations were performed in ArcView GIS [62]. The principal components extracted during the PCA and the identified cluster resulting from the cluster analysis were exported to ArcView GIS. The geographical distribution of principal components 1–4 across the CTs was cartographically displayed in four different maps, one map per principal component. The spatial distribution of the five identified clusters were also displayed in one map. All CTs belonging to one cluster were represented with the same colour resulting in a map with five different colours each representing CTs belonging to the same cluster.

## Results

### Correlations

All the observed correlations were in the expected directions (Table 2). For instance, neighbourhoods with high proportions of low-income earners were more likely to have high percentages of non-official language population (r = 0.62), single-parent families (r = 0.8), population living alone (r = 0.6), and high population density (r = 0.6). Moreover, neighbourhoods with high percentage of visible minority population also tended to have high proportions of new immigrants (r = 0.69), low-income persons (r = 0.61), and low percentage of owner-occupied dwellings (r = -0.61). Additionally, neighbourhoods with high percentage of population with less than Grade 9 education, tended to have low median income (r = -0.65), and a high percentage of population receiving government transfer income (r = 0.7).

**Table 2:** Correlation matrix of variables used in multivariate analyses of socioeconomic and demographic variables in Hamilton neighbourhoods, 2004. Numbers not in brackets are pair-wise correlation coefficients whereas those in brackets are p-values. A: Persons with less than grade 9 education; B: New immigrants; C: Visible minority; D: Aboriginal persons; E: Median income; F: Government transfer income; G: Low-income persons; H: Non-official language population; I: Unemployment rate; J: Dwelling value; K: Owner-occupied dwellings; L: Population aged under 20 years; M: Population aged 65 years or older; N: Single-parent families; O: Married population; P: Population living alone; Q: Internal migrants R: Population Density

	**A**	**B**	**C**	**D**	**E**	**F**	**G**	**H**	**I**	**J**	**K**	**L**	**M**	**N**	**O**	**P**	**Q**	**R**
**A**	1.00																	
**B**	0.12 (0.166)	1.00																
**C**	0.28 (0.001)	0.69 (<.001)	1.00															
**D**	0.24 (0.006)	-0.04 (0.623)	0.12 (0.176)	1.00														
**E**	-0.65 (<.001)	-0.35 (<.001)	-0.48 (<.001)	-0.59 (<.001)	1.00													
**F**	0.70 (<.001)	0.31 (<.001)	0.37 (<.001)	0.11 (0.229)	-0.68 (<.001)	1.00												
**G**	0.52 (<.001)	0.50 (<.001)	0.61 (<.001)	0.22 (0.013)	-0.74 (<.001)	0.82 (<.001)	1.00											
**H**	0.66 (<.001)	0.59 (<.001)	0.62 (<.001)	0.04 (0.660)	-0.51 (<.001)	0.56 (<.001)	0.60 (<.001)	1.00										
**I**	0.49 (<.001)	0.34 (<.001)	0.44 (<.001)	0.11 (0.201)	-0.54 (<.001)	0.68 (<.001)	0.66 (<.001)	0.56 (<.001)	1.00									
**J**	-0.49 (<.001)	-0.45 (<.001)	-0.48 (<.001)	-0.19 (0.027)	0.68 (<.001)	-0.77 (<.001)	-0.82 (<.001)	-0.50 (<.001)	-0.54 (<.001)	1.00								
**K**	-0.25 (0.004)	-0.67 (<.001)	-0.61 (<.001)	-0.17 (0.054)	0.59 (<.001)	-0.62 (<.001)	-0.79 (<.001)	-0.50 (<.001)	-0.55 (<.001)	0.68 (<.001)	1.00							
**L**	-0.07 (0.426)	-0.12 (0.190)	0.10 (0.246)	0.09 (0.300)	0.14 (0.103)	-0.33 (<.001)	-0.16 (0.077)	-0.07 (0.413)	-0.12 (0.180)	0.28 (0.001)	0.38 (<.001)	1.00						
**M**	0.13 (0.132)	-0.06 (0.485)	-0.26 (0.003)	-0.22 (0.010)	-0.02 (0.859)	0.38 (<.001)	0.01 (0.948)	-0.01 (0.906)	0.06 (0.484)	-0.16 (0.072)	-0.11 (0.195)	-0.76 (<.001)	1.00					
**N**	0.46 (<.001)	0.31 (<.001)	0.43 (<.001)	0.18 (0.039)	-0.57 (<.001)	0.67 (<.001)	0.80 (<.001)	0.41 (<.001)	0.57 (<.001)	-0.75 (<.001)	-0.58 (<.001)	0.08 (0.394)	-0.12 (0.184)	1.00				
**O**	-0.32 (<.001)	-0.36 (<.001)	-0.44 (<.001)	-0.23 (0.007)	0.64 (<.001)	-0.67 (<.001)	-0.86 (<.001)	-0.39 (<.001)	-0.56 (<.001)	0.79 (<.001)	0.81 (<.001)	0.40 (<.001)	-0.10 (0.240)	-0.73 (<.001)	1.00			
**P**	0.10 (0.252)	0.34 (<.001)	0.29 (0.001)	0.08 (0.344)	-0.42 (<.001)	0.58 (<.001)	0.62 (<.001)	0.27 (0.002)	0.41 (<.001)	-0.59 (<.001)	-0.77 (<.001)	-0.72 (<.001)	0.44 (<.001)	0.33 (<.001)	-0.80 (<.001)	1.00		
**Q**	-0.33 (<.001)	0.16 (0.071)	0.14 (0.103)	0.35 (<.001)	-0.03 (0.698)	-0.19 (0.032)	0.02 (0.791)	-0.04 (0.671)	0.00 (0.962)	0.10 (0.251)	-0.25 (0.004)	-0.03 (0.756)	-0.24 (0.005)	-0.11 (0.231)	-0.16 (0.076)	0.24 (0.006)	1.00	
**R**	0.18 (0.038)	0.42 (<.001)	0.43 (<.001)	0.11 (0.219)	-0.41 (<.001)	0.44 (<.001)	0.60 (<.001)	0.32 (<.001)	0.36 (<.001)	-0.62 (<.001)	-0.67 (<.001)	-0.28 (0.002)	0.05 (0.553)	0.48 (<.001)	-0.64 (<.001)	0.59 (<.001)	-0.02 (0.821)	1.00

### Principal components analysis

Only the first five principal components, that had eigenvalues greater than 1, were extracted (Table 3). The first PC is associated with the largest eigenvalue. This PC is a linear combination of the variables that account for the highest variability (46.1%) in the data. The second PC explains the highest variability not accounted for by the first PC while the third PC explains the largest variability not accounted for by the first two PC and so on. The first five PCs together accounted for a total of 84.23% of the total variation in the data (Table 3).

**Table 3:** Component loadings of socio-economic and demographic factors in the Hamilton, Ontario, Canada (2004)

**Variable**	**Principal component**					**Uniqueness**
	**1**	**2**	**3**	**4**	**5**	
Persons with <grade 9 education	0.575	0.176	0.659	-0.128	0.292	0.103
New immigrants	0.583	0.164	-0.337	0.539	0.243	0.171
Visible minority	0.643	0.419	-0.236	0.361	0.159	0.200
Aboriginal percentage	0.246	0.333	-0.165	-0.788	0.238	0.123
Median income	-0.790	-0.200	-0.125	0.378	-0.203	0.136
Government transfer income	0.847	-0.153	0.372	-0.053	0.017	0.118
Low income	0.941	0.110	0.018	-0.026	-0.144	0.081
Non-official language pop	0.675	0.255	0.197	0.342	0.424	0.143
Unemployment rate	0.721	0.103	0.165	0.036	0.064	0.437
Average dwelling value	-0.874	0.044	-0.103	0.042	0.203	0.181
Owner-occupied dwellings	-0.867	0.099	0.354	-0.096	0.000	0.104
Population under 20 years old	-0.324	0.867	0.089	0.045	-0.228	0.082
Population 65 years or older	0.139	-0.861	0.282	0.014	0.217	0.113
Single-parent families	0.759	0.252	0.160	-0.073	-0.437	0.138
Married percent	-0.875	0.148	0.190	0.172	0.251	0.084
Percent living alone	0.711	-0.552	-0.337	-0.075	-0.010	0.071
Percent of internal migrants	0.036	0.112	-0.790	-0.280	0.292	0.198
Population density	0.681	-0.113	-0.225	0.101	-0.325	0.357

Eigenvalue	8.30	2.42	1.92	1.47	1.06	-
Percentage of variation explained	46.10	13.42	10.69	8.14	5.88	-
Cumulative % of variation explained	46.10	59.52	70.35	78.35	84.23	-

The first Principal Component is the most important since most (12) of the variables loaded heavily on it (Table 3). The component loadings measure the relationships of the socioeconomic and demographic variables with each of the PCs. The values of the loadings range from -1 to 1. The uniqueness values of almost all the variables were relatively low with the highest being 0.437 and the lowest 0.071. Uniqueness is the percentage of variance for a variable that is not explained by the PCs. For instance, almost all the variations for low-income, population under 20, percent married and percent living alone are explained by the five PCs (Table 3).

**Figure 1:**
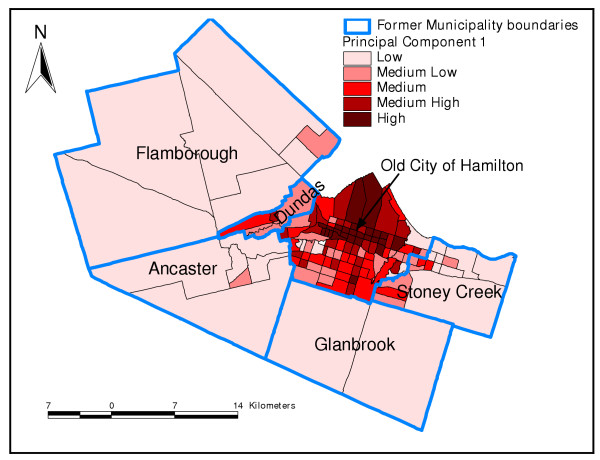
**Principal component 1**. Spatial distribution of first principal component extracted in the principal components analysis of socio-economic factors in Hamilton, Ontario, Canada (2004)

Principal component 1 is mainly an economic status component but also had a social component. Neighbourhoods (CTs) with high values of this PC had high proportions of aboriginal people, low median income, high percentage of low-income earners, relatively high percentage of individuals receiving government transfer income, high unemployment rates, low dwelling values, a high percentage of single-parent families, few owner-occupied dwellings, few married people, high percentage of persons living alone and high population density (Figure 1 and Table 3). These neighbourhoods could be described as high risk neighbourhoods because they had high values of most of the undesirable socioeconomic determinants of health.

**Figure 2:**
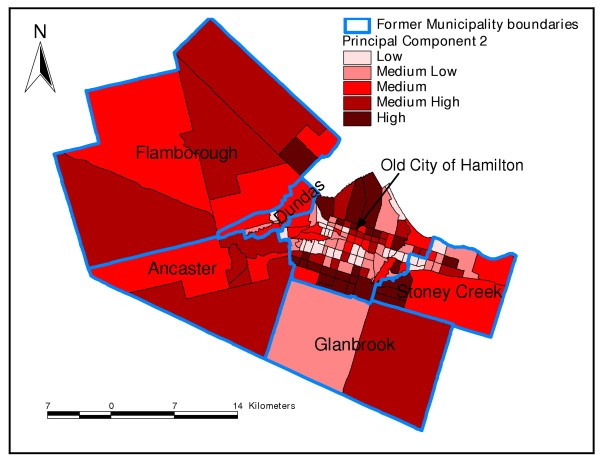
**Principal component 2**. Spatial distribution of second principal component extracted in the principal components analysis of socio-economic factors in Hamilton, Ontario, Canada (2004)

**Figure 3:**
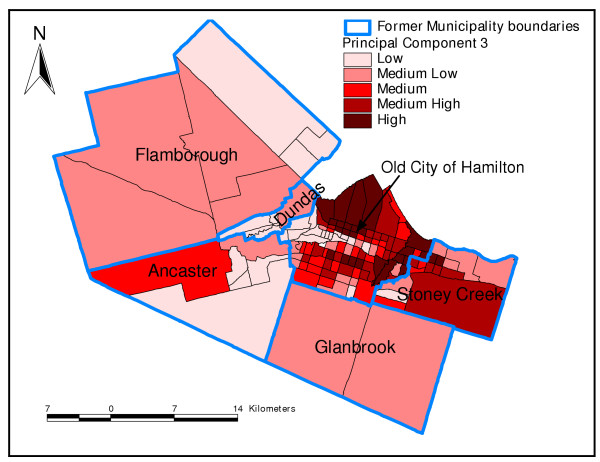
**Principal component 3**. Spatial distribution of third principal component extracted in the principal components analysis of socio-economic factors in Hamilton, Ontario, Canada (2004)

**Figure 4:**
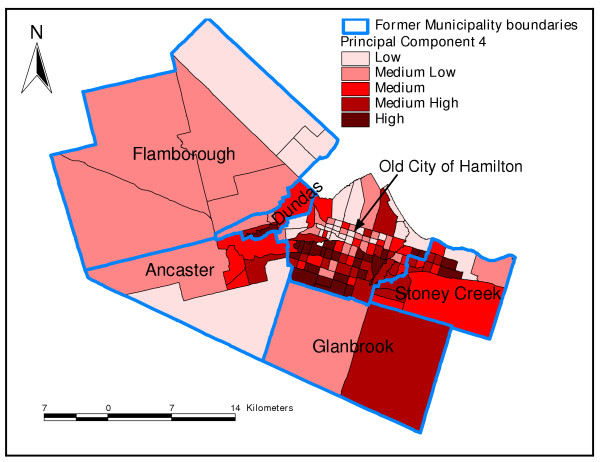
**Principal component 4**. Spatial distribution of fourth principal component extracted in the principal components analysis of socio-economic factors in Hamilton, Ontario, Canada (2004)

Principal component 2 was generally a demographic component. Neighbourhoods with high values of PC 2 had high percentages of children less than 20 years of age, but low percentages of seniors (Figure 2 and Table 3). Neighbourhoods with high values of PC 3 had high percentages of population with less than grade 9 education but low percentages of internal migrants (Table 3 and Figure 3). Finally, neighbourhoods that had high values of the fourth PC, an immigration and aboriginal status component, had high percentage of new immigrants but low population of people of aboriginal origin (Figure 4 and Table 3). The last PC (PC 5) did not load highly on any of the variables but was extracted because its eigenvalue was slightly higher than 1 (Table 3).

### Cluster analysis

**Figure 5:**
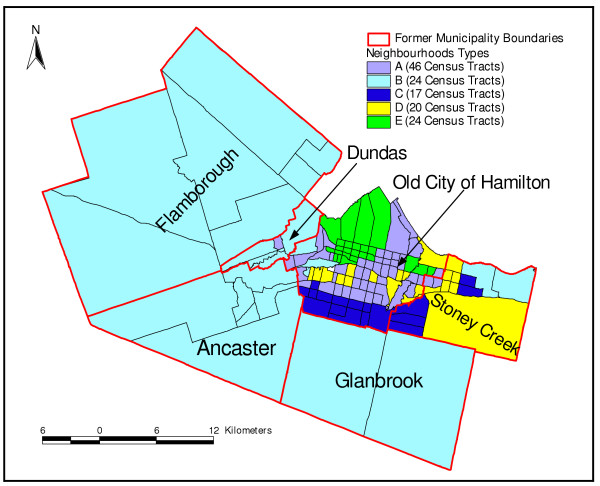
**Neighbourhood types**. Spatial distribution of identified neighbourhood types in Hamilton Ontario, Canada (2004)

Figure 5 shows the geographical distribution of the identified neighbourhood types (clusters). The detailed descriptive statistics of the socioeconomic and demographic factors for each of the neighbourhood types compared to the entire city of Hamilton is presented in Table 4. Statistical significance tests were performed to compare the characteristics of each of the neighbourhood types with the Hamilton average. Measures that were significantly different from the Hamilton average are described as high or low otherwise they are described as medium.

Neighbourhood Type A is primarily located within an inner ring surrounding the downtown core. These neighbourhoods can be described as "mature" areas (i.e., many seniors) with some indications of transition (i.e., neighbourhood turnover with arrival of new immigrants). Neighbourhood type A consists of 46 census tracts. A high population density, percentage of seniors and low-income earners, and a medium percentage of new immigrants characterize this neighbourhood type. It also has a high percentage of single-parent families, low dwelling values and a medium percentage of persons not able to speak English or French. Approximately 32.7% of Hamilton residents live in this neighbourhood type.

Neighbourhood Type B includes high economic status neighbourhoods in low-density rural or suburban environments. Since it covers the largest geographical area, its low population density has a great impact on the overall population density of Hamilton. These areas constitute the geographic periphery of the city, forming an outer ring from east to west. Neighbourhood type B is comprised of 24 census tracts and has approximately 23% of Hamilton residents. It has high median income, dwelling values, and percentage of owner-occupied dwellings and a medium percentage of seniors. This neighbourhood type also has a low percentage of new immigrants, single-parent families, and individuals with less than grade 9 education.

Neighbourhood Type C represents a relatively high economic status neighbourhoods within a more urban environment. Unlike Neighbourhood Type B, an area similar in income and dwelling value levels, this NT has a relatively high percentage of visible minority groups. It consists of 17 census tracts and is characterized by high median income, and dwelling values. In addition, this NT has few seniors and persons living alone, but a medium percentage of individuals who cannot speak either English or French. It also has high dwelling values and population density. Approximately 16.9% of Hamilton residents live in this type of neighbourhood.

Neighbourhood Type D depicts a "mature" urban area with a high percentage of seniors and owned dwellings. It has a relatively low percentage of low-income earners and a high percentage of individuals with less than grade 9 education. In addition, this neighbourhood type has few recent immigrants and internal migrants, low unemployment rate, high percentage of owner-occupied dwellings and medium percentage of persons not able to speak English or French. It is composed of 20 census tracts and 9.6% of Hamilton residents live in this type of neighbourhood.

Neighbourhood Type E constitutes the inner city core and a few areas scattered in the inner ring around the core, and is comprised of 24 census tracts. It has a high prevalence of low-income earners, new immigrants, visible minority groups, and persons with less than grade 9 education. It also has many single-parent families, those receiving government transfer income and high unemployment rate. Approximately 17.7% of Hamiltonians live in these neighbourhoods. Note that the sum of the population percentages of the groups is not 100% because one census tract was not included in the analysis because of missing data.

## Discussion

This study has used multivariate techniques to characterize neighbourhoods based on differences and/or similarities of their socioeconomic and demographic characteristics. The positive correlation between single-parenthood and low-income is consistent with observations from other studies that single-parents generally tend to spend more time in low-income neighbourhoods compared to childless couples and unattached individuals [63]. Moreover, it has also been reported that single-parenthood is common among socially disadvantaged groups and compounds social disadvantage [64]. In low socioeconomic neighbourhoods, people experience barriers in creating and benefiting from social capital, leading to social exclusion. The societal costs of social exclusion are lack of cohesion, higher crime rates, increased pressure on societal services and the stigma associated with particular neighbourhoods. Social exclusion is especially a problem in neighbourhoods with high unemployment rates, low-incomes, poor housing, etc., all of which combine to create a vicious cycle of poverty, low social capital and increased health risks [65].

The negative correlation between housing ownership and visible minority has been reported in other studies [66]. In addition, the observed positive correlation between visible minority and low-income has also been reported in other Canadian studies which reported that visible minority Canadians (people of colour) experience persistent income gap, above average levels of living on low-income and higher levels of unemployment [67]. The high negative correlation of low-income and housing ownership is not surprising and is in agreement with observations by Anderson and co-workers [68] who noted inadequate supply of affordable housing for low-income families and the increasing spatial segregation of some households by income, race, ethnicity, or social class into "unsafe neighbourhoods". Moreover, when affordable housing is not available to low-income households, family resources needed for food, medical or dental care, and other necessities are diverted to housing costs leading to the concept of "concentrated poverty" in certain neighbourhoods [68].

The low uniqueness values of the PCA imply that the five PCs appropriately represent the socioeconomic and demographic variables included in the analysis. Uniqueness values higher than 0.6 are considered high [69]. The advantage of using either PCA or cluster analysis in this kind of study is that they allow incorporation of many variables in the characterization of neighbourhoods. Therefore, from a population health planning perspective, they provide a better understanding of neighbourhood characteristics compared to representations based on only one variable. This is because the health of a population is determined by several socioeconomic, demographic and health care service factors and therefore analyses that incorporate only one variable would provide insufficient information for population health planning purposes.

Choice of the unit of analysis is critical in these kinds of analyses due to the modifiable areal unit problem (MAUP) since choice of a different and/or inappropriate unit could lead to quite different results [70,71]. As has been pointed out by Ross and coworkers (2004) [72], it is more meaningful to use 'naturally' defined neighbourhoods, rather than arbitrary geostatistical or political units since the distribution of population characteristics or health outcomes may not necessarily follow these arbitrary/political boundaries. Ross and co-workers compared the performance of census tracts to more 'natural' neighbourhoods and found very similar results and concluded that census tracts, used as proxies of neighbourhoods in our study, are good proxies for natural neighbourhood boundaries [72].

In this study, the results of the PCA were generally similar to those of cluster analysis since the distribution of areas identified as high risk by PC1 tended to follow similar spatial patterns as the high risk areas identified by cluster analysis. Both methods are therefore useful in identifying neighbourhood socioeconomic characteristics that would enhance health planning. However, as has been pointed out by Luginaah and co-workers [49], interpretation of the results of PCA is difficult due to its subjective nature and the fact that as many maps as number of principal components have to be produced. This makes cluster analysis methodology better for these purposes. Moreover, cluster analysis allows computation of statistics for each of the clusters (neighbourhood types) making the methodology much more objective than PCA.

Similar to the pattern seen in other industrial North American cities [28,73-75] most of the high risk neighbourhoods in this study (i.e. with high percentage of low-income earners, low educational attainment, etc), were located in the downtown core with the risk decreasing towards the suburban environments. The observed diverse neighbourhood socioeconomic characteristics may imply great variability in the health needs of the different population subgroups living in the different neighbourhoods since the conditions in which people live strongly influence their health. Health inequalities are produced by the clustering of several of these socioeconomic risk factors [76]. Therefore, populations living in different neighbourhood types differ in the type and number of socioeconomic risk factors to which they are exposed [77,78]. Although it is obvious that neighbourhood type E has the lowest socioeconomic status and highest risk while neighbourhood type B has the highest status and lowest risk, the intent of this study was not merely to classify the neighbourhoods based on economic status. Rather, this study was intended to generate neighbourhood socioeconomic information on which needs-based health planning and service delivery can be based. There is benefit in targeting improvement strategies to materially and socially deprived groups [79].

### Future directions and potential applications

The current study is the first of a series of projects designed to investigate neighbourhood health inequalities and provide information to foster health planning with a view to reducing health inequities. The identified neighbourhood clusters will be used, in subsequent studies, as units of analyses in investigating equity in health status, access and utilization of health services. Additionally, the identified of neighbourhood characteristics are expected to provide useful information on which health planning decisions will be based in order to:

1) Identify population health needs at the neighbourhood level

2) Assess health service utilization patterns across neighbourhoods and compare these with neighbourhood population characteristics and needs

3) Create geographic boundaries for the integrated delivery of social and community health care services

4) Allow for the development of strategies tailored and responsive to the unique characteristics and needs of each neighbourhood.

5) Enhance the use of empirical data for local advocacy for marginalized and under-served neighbourhoods and other populations in need.

Incorporation of the differences in neighbourhood socioeconomic characteristics in population health planning decisions such as decisions on funding allocation to community health agencies will help ensure that health planning strategies are best tailored to address the unique needs of each population. This is because a "one-size-fits-all" planning approach is neither efficient nor practical due to the different socioeconomic and demographic characteristics of the different neighbourhoods. It is expected that inclusion of neighbourhood socioeconomic and demographic characteristics in population health planning will provide health planners with more evidence to guide needs-based decisions that would be more appropriate for the socioeconomically diverse neighbourhoods. Therefore, it is hoped that the results of these analyses will be useful in ensuring that planning is tailored to the unique needs of the different neighbourhood population groups. For instance, neighbourhood types A and D have very similar median incomes and therefore if income was the only variable used to characterize the neighbourhoods, they would be treated similarly. However, the rest of the characteristics of these neighbourhood types are different. For example, neighbourhood type D has a much lower percentage of new immigrants, visible minority population and single-parent families than neighbourhood type A. Moreover, there are significantly more owner occupied dwellings in neighbourhood type D than A. The implication is that these neighbourhoods have potentially different challenges and health needs. If only median income was used (as is most often done) to classify the neighbourhoods, the two NTs would inevitably erroneously be treated as similar. Planning strategies based on such single variable analysis may not be appropriate since the strategies would not be tailored to the unique characteristics and therefore needs of the NTs.

### Conclusion

In this study, we have used multivariate techniques to identify unique neighbourhood characteristics and classify the neighbourhoods into groups with similar characteristics. Since the identified neighbourhood types are homogeneous with respect to the broad determinants of health, they offer potentially excellent opportunities for health planners and service providers to understand the characteristics and potential health needs of the different neighbourhoods and therefore better plan for them. Through continuous monitoring of health information across these neighbourhoods, health planners, service providers and policy makers could better make decisions based on knowledge of the local communities.

## Authors' contributions

AO was involved in the study design, execution and writing up of the draft and final copies of the manuscript. RW conceived the need for the study, was involved in the study design and preparation of the manuscript. ME, SB, BH, JE, and TA participated in the study design, guiding implementation and preparation of the draft of the manuscript.

**Table 4:** Summary statistics of socio-economic and demographic features of identified neighbourhood types in Hamilton, 2004. Data source: Statistics Canada, 2001 census. NT = Neighbourhood type. The sum of the populations from the 5 neighbourhood types is not 490,268 due to missing data in one census tract.

**Variable**	**Means**					
	
	**Hamilton**	**NT A**	**NT B**	**NT C**	**NT D**	**NT E**
Persons with <grade 9 education (%)	10.3	11.3	4.7	9.2	13.7	16.9
New immigrants (%)	3.2	3.4	0.9	2.7	1.2	6.6
Visible minority (%)	10.9	9.4	4.2	14.4	5.6	20.7
Aboriginal persons (%)	2.2	2.6	1.0	1.6	3.0	4.1
Median income ($)	22927	22262	30351	25958	22837	16250
Government transfer income (%)	12.1	15.9	7.3	8.3	13.4	23.2
Low income (%)	19.8	21.7	5.6	12.7	10.7	42.4
Persons not speaking English or French (%)	1.8	1.6	0.33	1.6	1.6	4.4
Unemployment rate (%)	6.4	5.7	2.2	4.2	3.5	10.1
Average dwelling value ($)	166783	125271	224342	173801	162157	89174
Owner-occupied dwellings (%)	65.2	61.9	87.4	81.8	86.7	34.7
Population under 20 years old (%)	26.1	23.5	27.5	33.6	21.2	23.6
Population 65 years or older (%)	14.3	17.2	13.2	7.2	21.0	14.3
Single-parent families (%)	16.6	19.9	9.5	15.4	10.7	25.7
Married (%)	51.7	47.3	60.9	58.9	58.5	35.7
Live alone (%)	10.3	13.3	5.4	2.9	8.7	21.1
Internal migrants (%)	10.6	9.2	14.5	7.7	6.9	12.2
Population Density (No. of persons per Km^2^)	438.9	3627.4	357.1	2543.2	2352.1	5706.0
Population (count)	490268	160438	112875	82976	47137	86683
